# A transcriptomic dataset evaluating the effect of radiotherapy injury on cells of skin and soft tissue

**DOI:** 10.1016/j.dib.2022.107828

**Published:** 2022-01-14

**Authors:** Lipi Shukla, Stuart A. Lee, Mei R.M. Du, Tara Karnezis, Matthew E. Ritchie, Ramin Shayan

**Affiliations:** a O'Brien Institute Department, St Vincent's Institute for Medical Research, Fitzroy, Victoria 3065, Australia; b Epigenetics and Development Division, The Walter and Eliza Hall Institute of Medical Research, Parkville, Victoria 3052, Australia; c Department of Plastic Surgery, The Alfred Hospital, Prahran, Victoria 3004, Australia; d Department of Plastic Surgery, St. Vincent's Hospital, Fitzroy, Victoria 3065, Australia; e Department of Medicine, St. Vincent's Hospital, University of Melbourne, Fitzroy 3065, Australia

**Keywords:** Gene expression, Radiotherapy injury, Skin

## Abstract

Radiotherapy injury to cells of the skin and subcutaneous tissue is an inevitable consequence of external beam radiation for treatment of cancer. This sublethal injury to normal tissues plays a significant role in the development of fibrosis, lymphedema, impaired wound healing, and recurrent infections. To elucidate the transcriptional changes that occur in cells of the skin and soft tissues after radiotherapy injury, we performed genome-wide RNA-sequencing comparing irradiated cells (10Gy) with non-irradiated (0Gy) controls in normal human dermal fibroblasts, normal human keratinocytes, human microvascular endothelial cells, human dermal lymphatic endothelial cells, pericytes and adipose derived stem cell populations. These data are publicly available from the Gene Expression Omnibus database (accession number GSE184119). Further insights can be gained by comparing the mRNA signatures arising from radiation injury derived from these data to publicly available signatures from other studies involving similar or different tissue types. These global targets hold potential for manipulation to mitigate radiotherapy soft tissue injury.

## Specifications Table

**Table:** 

Subject	Biological sciences, Omics: Transcriptomics
Specific subject area	An invitro model to determine the transcriptional changes resulting from sublethal radiotherapy injury to cells that constitute the skin and subcutaneous tissues.
Type of data	Unmapped fastq Gene-level counts
How the data were acquired	Six different cell types were subjected to different doses of radiotherapy. RNA was then extracted from tissue culture and purified. Samples were tested for purity and quality before undergoing RNA sequencing using an Illumina HiSeq.
Data format	Raw fastq files Summarised gene-level count files (.txt)
Description of data collection	RNA sequencing was performed under normal conditions on cells in duplicate or triplicate under no treatment (0Gy) or radiotherapy treatment (10Gy single dose). A separate group of adipose derived stem cells and lymphatic endothelial cells were treated with a 5 × 2Gy fractionated dose regime of radiotherapy. Two biological replicates of each cell-type/treatment combination are available.
Parameters for data collection	Key and supportive cells of the lymphatic and blood vessels, cells creating the structure of skin and cells dictating the regenerative potential of subcutaneous fat adipose-derived stem cells were chosen. Sublethal radiotherapy doses replicated therapeutic doses used in clinical practice and had been calibrated from a dose response curve, allowing the highest sublethal effect that could be standardized across cell types.
Data source location	• Institution: Australian Genome Research Facility (AGRF) • City/Town/Region: Parkville, Victoria • Country: Australia
Data accessibility	Repository name: Gene Expression Omnibus (GEO) Data identification number: GSE184119 Direct URL to data: https://www.ncbi.nlm.nih.gov/geo/query/acc.cgi?acc=GSE184119 Analysis scripts and gene signatures from the analysis of these data are available from https://github.com/mei-du/radiation. Tables from the differential gene expression analysis are also available with the article (Supplementary Files 1 and 2).
Related research article	L. Shukla, *et al.* Therapeutic Reversal of Radiotherapy Injury to Pro-fibrotic Dysfunctional Fibroblasts In Vitro Using Adipose-derived Stem Cells. *Plastic and Reconstructive Surgery Global Open* 8 (2020) e2706. 10.1097/GOX.0000000000002706

## Value of the Data

• These data are useful as they allow us to measure the effects of radiotherapy (RTX) on gene expression at the level of the individual cell types that constitute skin to define a radiation gene signature across all the different cell types profiled.
• These data could be used to help clinicians understand and address the adverse effects of RTX on normal soft tissues.
• Further insights can be gained by comparing the radiation signatures derived from these data to publicly available signatures from other studies involving similar or different tissue types.

## 1 Data Description

The dataset consists of 28 samples from 6 different cell types, including 2 biological replicates. Each replicate was subjected to a single dose of RTX (10Gy) or no RTX (0Gy, control) and for adipose derived stem cells (ADSC) and lymphatic endothelial cells (LEC), a separate 5 × 2Gy fractionated dose. Files relevant to each sample are raw RNA-seq fastq files and processed gene-level count text files displaying a gene identifier, gene length and normalised read count. These files are accessible via GEO and named by cell type, RTX dosage and replicate number.

The provided GitHub repository hosts files relevant to exploratory data analysis including quality control and preprocessing of count data, differential expression analysis, gene set analysis and figures. A project file (radiation.Rproj), README file (README.md) and lockfile (renv.lock), along with “activate.R” in the “renv” folder are provided to facilitate reproduction of the analysis using R [1].

In the “analysis” folder, numbered R scripts detail steps of the analysis, complimented by various RData and R objects in the “data” folder. Normalisation steps are shown in “01-normalisation.R” in the “analysis” folder. Count data are contained in “counts.rda” and processed and transformed count data are contained in “processed_counts.rds” in the “data” folder. The transformed counts of one pericyte sample (PERI1_10Gy) were merged as the sample libraries were split across three sequencing lanes which led to 30 fastq files for the 28 samples.

**Fig. 1:**
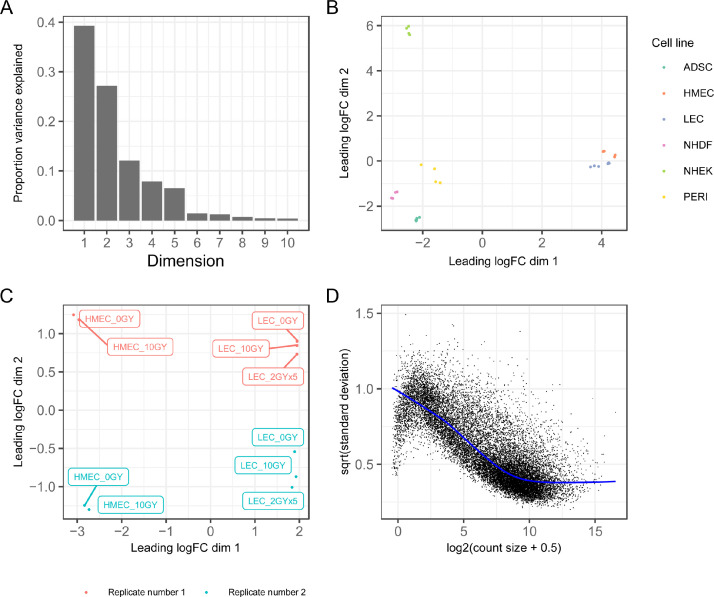
**Figures summarising exploratory data analysis.** (**A)** The proportion of variance explained by each dimension from the multidimensional scaling (MDS) analysis. (**B)** MDS plot of all samples from the experiment, coloured by cell line. (**C)** MDS plot of the LEC and HMEC cell lines. (**D)** Mean-variance trend in expression estimated by voom, with points representing genes.

This figure was generated using “02-eda.R” in the “analysis” folder, and its file (figure-01.png) is contained in the “figures” folder in the GitHub repository. The first and second dimension explain 39 and 27 percent of the variation in the data, respectively (Fig. 1A). There is clear separation shown in the MDS plots between the different cell types across dimensions 1 and 2 (Fig. 1B) and replicate number (Fig. 1C, dimension 2) when considering a subset of the data. The contrast matrix, and combined sample-specific quality weights [2] and *voom* [3,4] precision weights matrix based on the mean-variance relationship (Fig. 1D), were saved to “contrasts.rds” and “voom.rds” in the “data” folder, respectively.

**Fig. 2:**
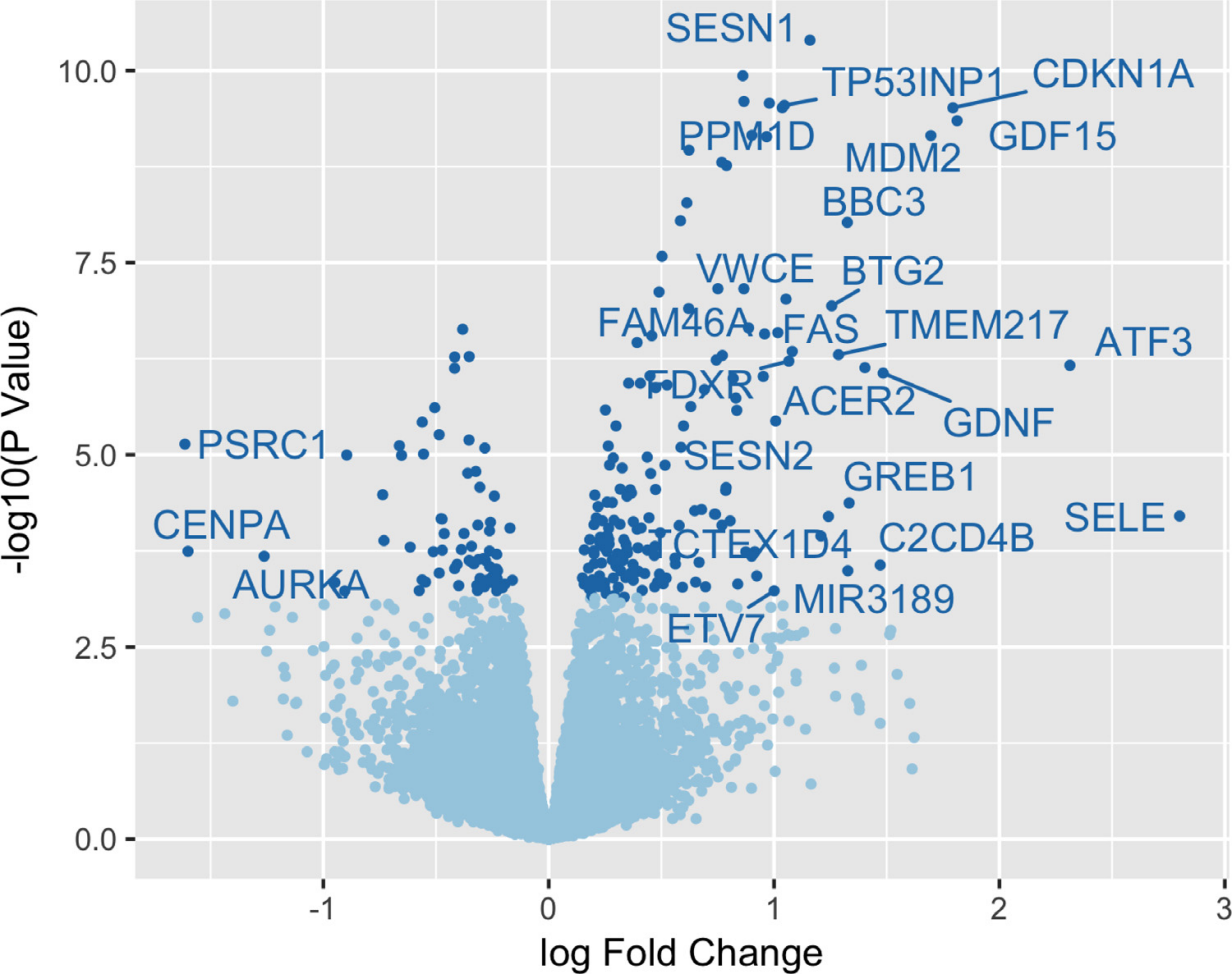
**Summary of the differential expression analysis.** A volcano plot of the differential gene expression analysis between full radiotherapy treatment and controls (10Gy vs. 0Gy).

Fig. 2 is a volcano plot of differential gene expression between full radiotherapy treatment and controls (10Gy vs. 0Gy), created using “03-de.R” from the “analysis” folder of the GitHub repository. The x-axis represents the log_2_(fold-change) in gene expression and the y-axis represents the negative log_10_(P-value) of a gene being differentially expressed. The dark blue points indicate a differentially expressed gene using a false discovery rate (FDR) cut-off of 0.05. Labelled genes are significantly differentially expressed genes with a log_2_(fold-change) greater than 1. The file for this figure (figure-02.png) is in the “figures” folder . Between 10Gy RTX and 0Gy control treatments, there were 243 differentially expressed genes. Of these, 73 genes were down regulated in the 0Gy control cells, while 170 upregulated when exposed to 10Gy radiotherapy treatment (Fig. 2). Numerous genes correlated well with the postulated pathological processes and clinical tissue side effects observed in patients post-RTX. Globally significantly altered RNA candidates across all proliferating cell types include ATF3, SELE, CDKN1A, GDF15 (growth differentiation factor 15) and MDM2 which all play a role in cell cycle regulation/arrest, DNA damage response and repair by exerting anti-oxidant, anti-proliferative and pro-apoptotic effects. Further pathway analysis demonstrated that these alterations consist of significant enrichment of genes in the p53 signalling pathway.

There were no differentially expressed genes between cells treated with fractionated doses (2Gy x 5) of RTX and the single dose (10Gy) of RTX. We found 70 differentially expressed genes for the comparison between the cells treated with fractionated RTX and control (0Gy) treatment, with 6 genes down regulated in control cell types, compared to 64 genes upregulated in the fractionated dosage group.

The top-ranked differentially expressed genes from the linear model fitted using all cell types (Supplementary File 1: top_tables_by_contrast.csv) and another file listing such genes from the model fitted using LEC data (Supplementary File 2: lec_top_tables.csv) are contained in the “analysis” folder and also provided as supplementary files. The top-ranked enriched gene ontology (GO) terms (go_terms_lec.csv) and KEGG pathways (kegg_pathways_lec.csv) for differentially expressed genes derived from the LEC data are available in the “analysis” folder.

## 2 Experimental Design, Materials and Methods

### 2.1 Sample information

The effect radiotherapy has on gene expression was measured in 6 different human cell types: 1) normal dermal fibroblasts (NHDF) (C -12302 PromoCell Germany) cultured in DMEM 4.5 g/L Glucose with Ultraglutamine (Lonza, Switzerland), 10% fetal calf serum (SAFC Biosciences, USA), 1% antibiotic and sodium pyruvate (Gibco, USA); 2) normal human epidermal keratinocytes (NHEK) (C-12003 PromoCell) cultured in Keratinocyte Basal growth medium with growth factors (PromoCell); 3) human placental pericytes (PC) (C-12980 PromoCell) cultured in pericyte basal growth medium and supplemental kit (PromoCell); 4) microvascular blood endothelial cells (HMEC (CC-2543 Lonza) cultured in endothelial cell basal medium 2 and supplemental mix (Lonza); 5) lymphatic endothelial cells (LEC) (C-12217 PromoCell) cultured in endothelial cell basal medium MV2 and supplemental mix (PromoCell) and 6) adipose derived stem cells (ADSCs) isolated as per methods described by Zuk et al. [5], cultured in the same media as NHDF. Cell cultures were established from cryopreserved commercially available vials or fresh tissue isolation (as detailed above) and incubated in 37°C, 5% CO_2_ conditions in tissue culture treated T75 flasks (CELLSTAR®, Germany). Each cell type was subjected to a single dose of RTX (10Gy) or no RTX (0Gy, control) at the Bio-resources Centre (Victoria, Australia) using a Gammacell® 40 Irradiator (Best® Theratronics, Canada). For the ADSC and LECs, fractionated doses (5 × 2Gy over a 48-hr period) were also administered. Doses were sublethal and calculated to replicate therapeutic doses used in clinical practice for soft tissue carcinomas and sarcomas. Two biological replicates of each cell-type/treatment combination were available, and the specific passage numbers of the samples analysed were: NHDF (passage 4 and 5), NHEK (passage 3 and 4), PC (passage 4 and 5), HMEC (passage 4 and 5), LEC (passage 3 and 5) and ADSC (passage 2 and 3).

### 2.2 RNA-seq sample preparation and sequencing

Standardized numbers of LECs, HMECs, NHDFs, NHEKs, PCs and ADSCs were plated in T75 flasks (CELLSTAR®) (as per manufacturer's instructions) and were irradiated once 80–90% confluence was achieved. RNA was extracted from tissue culture flasks at 4 h after irradiation or control treatment was completed using Qiazol® (QIAGEN, Germany) and purified with DNase and RNEasy® Plus Universal Kit (QIAGEN) as per manufacturer's instructions. Samples were then tested for purity and quality control using the Nanodrop Spectrophotometer (Thermo Fischer Scientific). Each sample was then transported to the Australian Genome Research Facility (AGRF) in Melbourne and underwent RNA sequencing (100 base pair single end) using Illumina HiSeq.

### 2.3 Quality control and data preprocessing

Sequenced reads were first mapped to the hg19 human reference genome using the R/Bioconductor package *Rsubread* [6,7] (version 1.10.5) with default parameters. Mapped reads were then assigned to individual genes, using the *featureCounts* [8] function with default settings. Genes were annotated using the *org.Hs.eg.db* [9] R/Bioconductor package (version 3.8.2). Read counts were processed with the R/Bioconductor packages *edgeR* [10,11] (version 3.8.1) and *limma* [12] (version 3.32.4). Counts were first transformed using the *cpm* function from *edgeR* to generate counts per million (cpm) for each gene to account for different library sizes. Genes were retained for further analysis if they had a baseline expression level of 0.5 cpm in at least three samples. Counts were normalized using the trimmed mean of M-values (TMM) method [13]. Multidimensional scaling (MDS) was performed on the transformed counts during exploratory data analysis.

### 2.4 Differential expression analysis

We modelled heteroscedasticity in the gene counts with *voomWithQualityWeights* from the *limma* package using radiotherapy treatment as a main effect (Fig. 1D), adjusting for cell type and replicate number. Observations that were more variable compared to others were down-weighted in the subsequent linear model analysis.

To investigate the global consequences of RTX across the selected cell subtypes, we used *limma* to fit a linear model and used empirical Bayes moderation of *t*-statistics [14] to assess differential expression between the contrasts of interest (10Gy vs. 0Gy, 10Gy vs. 2Gyx5, and 2GYx5 vs 0Gy. Gene set analysis was also performed for the same comparisons made on the LEC data using the *goana* and *kegga* functions of the *limma* package.

## Ethics Statements

This manuscript adheres to the Elsevier Ethics in publishing standards.

## CRediT authorship contribution statement

**Lipi Shukla:** Investigation, Validation, Writing – original draft. **Stuart A. Lee:** Formal analysis, Visualization, Writing – original draft. **Mei R.M. Du:** Formal analysis, Visualization, Data curation, Writing – original draft. **Tara Karnezis:** Supervision, Writing – review & editing. **Matthew E. Ritchie:** Formal analysis, Writing – review & editing. **Ramin Shayan:** Conceptualization, Resources, Supervision, Funding acquisition, Writing – review & editing.

## Declaration of Competing Interest

The authors declare that they have no known competing financial interests or personal relationships that could have appeared to influence the work reported in this paper.

## Appendix Supplementary Materials

Image, application 1

Image, application 2
