# Corrigendum to ‘A transcriptomic dataset evaluating the effect of radiotherapy injury on cells of skin and soft tissue’ Data in Brief 41 (2022) 107828

**DOI:** 10.1016/j.dib.2022.108358

**Published:** 2022-06-06

**Authors:** Lipi Shuka, Stuart A. Lee, Mei R.M. Du, Tara Karnezis, Matthew E. Ritchie, Ramin Shayan

**Affiliations:** a O'Brien Institute Department, St Vincent's Institute for Medical Research, Fitzroy, Victoria 3065, Australia; b Epigenetics and Development Division, The Walter and Eliza Hall Institute of Medical Research, Parkville, Victoria 3052, Australia; c Department of Plastic Surgery, The Alfred Hospital, Prahran, Victoria 3004, Australia; d Department of Plastic Surgery, St. Vincent's Hospital, Fitzroy, Victoria 3065, Australia; e Department of Medicine, St. Vincent's Hospital, University of Melbourne, Fitzroy 3065, Australia

The authors regret a typing error in the code used in the analysis of the dataset, which affected the values included in supplementary table ‘lec_top_tables.csv’. This table is relevant to the paragraph “The top-ranked differentially expressed genes from the linear model … another file listing such genes from the model fitted using LEC data (Supplementary File 2: lec_top_tables.csv)…”. The GitHub repository containing the relevant code and table has been updated with the correct results. Any further results are unaffected. The authors would like to apologise for any inconvenience caused.

